# Supplementary material for: COVID-19 Pandemic: Did Strict Mobility Restrictions Save Lives and Healthcare Costs in Maharashtra, India?
Source: Healthcare (Basel). 2023 Jul 24;11(14):2112. doi: 10.3390/healthcare11142112 (PMC10379405; doi:10.3390/healthcare11142112)
Supplement: Supplementary file 1 [file healthcare-11-02112-s001.zip › Ambade et al_2022_MH_COVID19_Annexure_SA.pdf]

## Annexure-A
## MEDD Data

**From MEDD 15 April 2020 Data**

| Age group | Midpoint | No. of deaths | F(x) | % of total deaths | % of deaths below 60 yrs. | | |
|---|---|---|---|---|---|---|---|
| upto 10 yrs | 5 | 0 | 0 | 0 | | | |
| 11 to 20 | 15 | 0 | 0 | 0 | | | |
| 21 to 30 | 25 | 5 | 125 | 2.92397661 | | | |
| 31 to 40 | 35 | 8 | 280 | 4.67836257 | | | |
| 41 to 50 | 45 | 26 | 1170 | 15.2046784 | | | |
| 51 to 60 | 55 | 52 | 2860 | 30.4093567 | 53.2163743 | | |
| 61 to 70 | 65 | 52 | 3380 | 30.4093567 | 83.625731 | **84** | **84% are premature deaths** |
| 71 to 80 | 75 | 22 | 1650 | 12.8654971 | | | |
| 81 to 90 | 85 | 5 | 425 | 2.92397661 | | | |
| 91 to 100 | 95 | 0 | 0 | 0 | | | |
| 101 to 110 | 105 | 1 | 105 | 0.58479532 | | | |
| | **Total** | **171** | **9995** | | | | |

**Estimated Mean (yrs.)** **58** **This is average age of death**
