# Supplementary material for: COVID-19 Pandemic: Did Strict Mobility Restrictions Save Lives and Healthcare Costs in Maharashtra, India?
Source: Healthcare (Basel). 2023 Jul 24;11(14):2112. doi: 10.3390/healthcare11142112 (PMC10379405; doi:10.3390/healthcare11142112)
Supplement: Supplementary file 1 [file healthcare-11-02112-s001.zip › Ambade et al_2022_MH_COVID19_Annexure_SB.pdf]

Annexure-B
Actual and Projected Cases

Date	Daily Cases*	No. of actual quarantined contacts@1:20 ratio	Projected Cases	No. of projected quarantined contacts@1:20 ratio
15-Apr-20	236	4720	371	7420
16-Apr-20	285	5700	418	8360
17-Apr-20	120	2400	469	9380
18-Apr-20	327	6540	526	10520
19-Apr-20	552	11040	589	11780
20-Apr-20	466	9320	659	13180
21-Apr-20	552	11040	736	14720
22-Apr-20	431	8620	824	16480
23-Apr-20	778	15560	924	18480
24-Apr-20	390	7800	1035	20700
25-Apr-20	811	16220	1158	23160
26-Apr-20	440	8800	1295	25900
27-Apr-20	522	10440	1450	29000
28-Apr-20	728	14560	1627	32540
29-Apr-20	597	11940	1820	36400
30-Apr-20	583	11660	2039	40780
1-May-20	1008	20160	2283	45660
2-May-20	790	15800	2557	51140
3-May-20	678	13560	2861	57220
4-May-20	1567	31340	3204	64080
5-May-20	984	19680	3580	71600
6-May-20	1233	24660	4014	80280
7-May-20	1216	24320	4496	89920
8-May-20	1089	21780	5038	100760
9-May-20	1165	23300	5638	112760
10-May-20	1943	38860	6310	126200
11-May-20	1230	24600	7076	141520
12-May-20	1026	20520	7924	158480
13-May-20	1495	29900	8863	177260
14-May-20	1602	32040	9914	198280
15-May-20	1576	31520	11106	222120
16-May-20	1606	32120	12453	249060
17-May-20	2347	46940	13925	278500
18-May-20	2005	40100	15599	311980

19-May-20	2078	41560	17458	349160
20-May-20	2161	43220	19573	391460
21-May-20	2345	46900	21908	438160
22-May-20	2940	58800	24556	491120
23-May-20	2608	52160	27484	549680
24-May-20	3041	60820	30789	615780
25-May-20	2436	48720	34417	688340
26-May-20	2091	41820	38589	771780
27-May-20	2190	43800	43179	863580
28-May-20	2598	51960	48398	967960
29-May-20	2682	53640	54182	1083640
30-May-20	2940	58800	60688	1213760
31-May-20	2487	49740	67889	1357780
**Total**	**64975**	**1299500**	**631891**	**12637820**

*Source: www.covid19.org accessed between 30th March 1st June 2020

Projected vs Actual cases and contacts for period 15 April-31 May 2020

		**Cases**	**Contacts**
Projected		631891	12637820
Actual		64975	1299500
