# Supplementary material for: COVID-19 Pandemic: Did Strict Mobility Restrictions Save Lives and Healthcare Costs in Maharashtra, India?
Source: Healthcare (Basel). 2023 Jul 24;11(14):2112. doi: 10.3390/healthcare11142112 (PMC10379405; doi:10.3390/healthcare11142112)
Supplement: Supplementary file 1 [file healthcare-11-02112-s001.zip › Ambade et al_2022_MH_COVID19_Annexure_SC.pdf]

COVID-19 Pandemic: Did harsh mobility restrictions save lives and cost in Maharashtra, India? Annexure-C

A. Fixed Cost Calculations

1000 0000

Total Allocation for COVID-19 in Maharashtra	In Cr.	Actual	Notes	Source
Initial Allocation	45 Cr.	450000000	As per media reports this is the initial allocation by state government to tackle COVID-19	https://www.livemint.com/news/india/coronavirus-update-maharashtra-allocates-rs-45-crore-to-fight-covid-19-as-cases-rise-to-39-11584368200723.html
Share of CMRF	23.82 Cr.	238200000	As per media reports state received 342 Cr. in CM Relief Fund for COVID-19 of which 23.82 was spent on COVID-19 related items	https://www.firstpost.com/health/maharashtra-cm-fund-receives-rs-342-cr-in-donations-till-mid-may-rs-23-cr-spent-on-covid-19-rs-55-cr-on-migrant-fare-reveals-rti-query-8437151.html

%35 share of SDRMF first installment which is allowed to spend on COVID-19 related expenditure	35% of 2148 Cr.	7518000000	35% of total first installment which was allowed to be spent on COVID-19 (25% on Measures for quarantine, sample collection and screening+10% on procurement of essential equipments/labs for response to COVID-19) this restrictions was later removed by the letter on 14 July 2020.and inter-category expenditure was allowed capping it to 35%.	https://economictimes.indiatimes.com/news/politics-and-nation/states-get-to-use-35-of-disaster-response-fund-for-covid-now/articleshow/77055801.cms?from=mdr
India COVID-19 Emergency Response and Health System Preparedness Package for Maharashtra (Until 10th September 2020)	726.38 Cr.	7263825615		
**Total**		**15470025615**	**1547.002562**	**Cr.**

B. SDRMF Calculations

In SDRMF, Center:State share is 75:25. Center released 1611 Cr. which is the first installment of its total share of 3222 Cr. For state share I have assumed Maharashtra has matched its 25% share to this first installment thus contributing 537 Cr. to SDRMF. The total first installment available under SDRMF becomes 2148 Cr. of which 35% i.e. 751.8 Cr. was allowed to be spent on COVID-19 related activities. This cap was later revised removing intra-category expenditure cap and allowing total expenditure up to 35% from the SDRMF letter issued on 14th July 2020. See the following table for calculation.

total allocation (Cr.)	Center Share (Cr.)	State share. (Cr.)	first installment center (Cr.)	first installment state (Cr.)	total first installment (Cr.)	35% of total first installment which was allowed to be spent on COVID-19 (25% on Measures for quarantine, sample collection and screening+10% on procurement of essential equipments/labs for response to COVID-19) this restrictions was later removed by the letter on 14 July 2020.and inter-category expenditure was allowed capping it to 35%.	10% of total share which was used towards COVID-19 medical expenditure	If we assume state matched central govt's share of first installment funds then 35% of first installment

4296	3222	1074	1611	537	2148	751.8	214.8	**751.8**

C. India COVID-19 Emergency Response and Health System Preparedness Package for Maharashtra Calculations

India COVID-19 Emergency Response and Health System Preparedness Package for Maharashtra disbursed as of 10th September 2020 (answer to unstarred question)

Items	Cost	Notes	Sources

CFX-96 Touch Real Time PCR Detection System @ Rs.14,86,800/-	10407600		100000
CFX96-IVD Real-Time PCR System @Rs.15,22,500/-	7612500		
Automated RNA Extraction @Rs.42,18,500/-	42185000		
COBAS 6800 @Rs.3,88,50,000/-	38850000		
RNA kit	113238072		
VTM kit	78776585		
RT-PCR kit	721803858		
N95 Maskes (distributed 29.26 lakh)	122892000	The price of N-95 procurement ranged between 42 to 63 Rs. Between March and May. We considered lowest price of 42 INR.	https://economictimes.indiatimes.com/industry/healthcare/biotech/healthcare/n95-mask-prices-rise-250-in-4-months-but-no-cap-yet/articleshow/76295506.cms
PPEKit(distributted 12.58 lakh)	943500000	considred 750 Rs. is cost Per kit. Range is (750-1000)	The costs of hospitalisation for COVID-19. https://economictimes.indiatimes.com/industry/healthcare/biotech/heal…e-costs-of-hospitalisation-for-covid-19/count/slideshow/75313849.cms

HCQ tablets(distributed 97.2 lakh)	29160000	Govt. procured tablets from Zydus Cadilla and Ipca Lab of which Zydus Cadila's price was available in media therefore their prices are considered which is Rs.3/tablet	https://theprint.in/health/govt-places-order-for-10-crore-hydroxychloroquine-tablets-with-ipca-labs-and-zydus-cadila/395248/
installed ventilators (3043)	1217200000	cost of one ventilator is calculated based on PM CARES FUND allocation for ventilators. GOI allocated 2000 Cr. INR for supply of 50000 ventilators. Thus cost of each ventilator = 4,00,000 INR. See calculations below.	https://pib.gov.in/PressReleseDetail.aspx?PMO=3&PRID=1633516
**Total Grant through Medical supplies**	3325625615		
	332.5625615	Cr.	
**Grant-in-aid**	**393.82**	Cr.	
**Total**	**726.38**	Cr.	
