# Supplementary material for: COVID-19 Pandemic: Did Strict Mobility Restrictions Save Lives and Healthcare Costs in Maharashtra, India?
Source: Healthcare (Basel). 2023 Jul 24;11(14):2112. doi: 10.3390/healthcare11142112 (PMC10379405; doi:10.3390/healthcare11142112)

COVID-19 Pandemic: Did harsh mobility restrictions save lives and cost in Maharashtra, India?

Annexure-D

Sensitivity Analysis input parameters and Tornado Diagram

Value Type	Parameter	Total Cost Difference USD (million)
	Days Spent in ICU (days)	
Low	4	2586.50
Base Value	7	2596.40
High	19	2636.02
	Days spent in non-ICU for ICU patients (days)	
Low	3	2586.12
Base Value	7	2596.40
High	10	2604.12
	Days spent in non-ICU for non-ICU patients (days)	
Low	4	2403.52
Base Value	14	2596.40
High	21	2731.42
	Days for quarantine for contacts (days)	
Low	7	1790.85
Base Value	14	2596.40
High	21	3401.96
	ICU Bed charges (INR)	
Low	7500	2595.00
Base Value	8825	2596.40
High	9000	2596.59
	Cost of PPE kit (INR)	
Low	500	2528.94
Base Value	750	2596.40
High	1000	2663.87

COVID-19 Pandemic: Did harsh mobility restrictions save lives and cost in Maharashtra, India?

Tornado diagram depicting net change in total cost difference (in million USD) from the base estimate of 2596.40 million USD

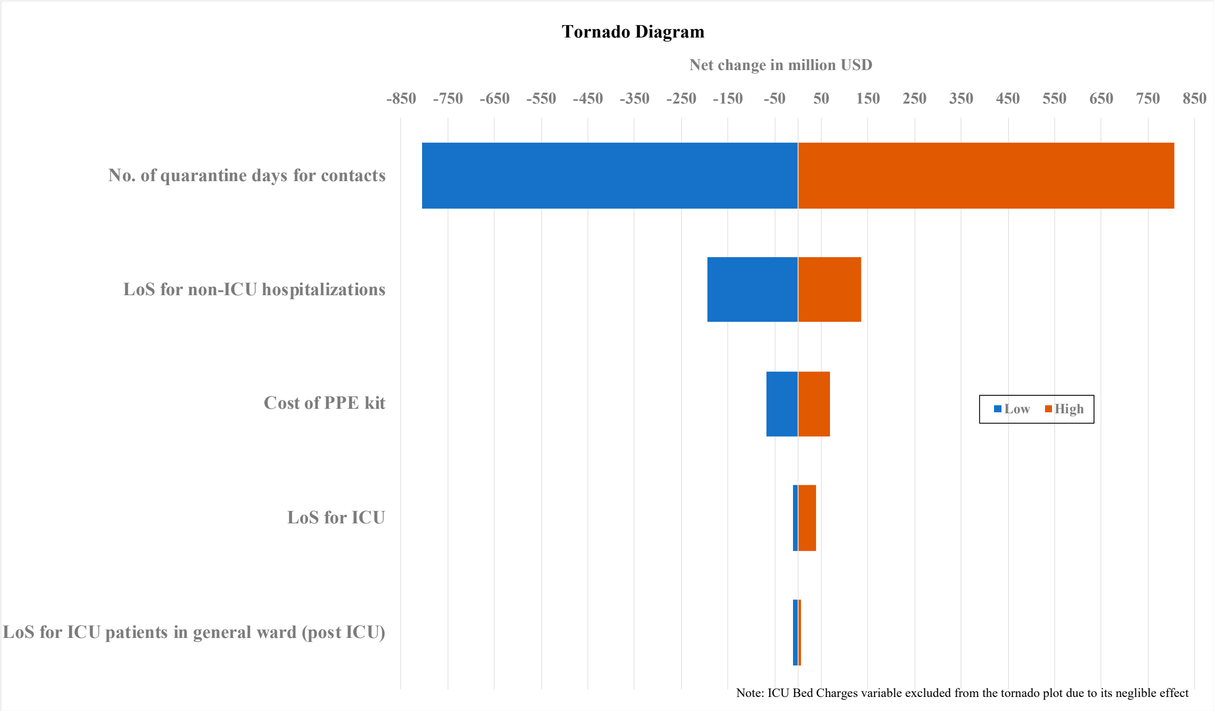

Supplement: Supplementary file 1 [file healthcare-11-02112-s001.zip › Ambade et al_2022_MH_COVID19_Annexure_SD.pdf]
