# Supplementary material for: COVID-19 Pandemic: Did Strict Mobility Restrictions Save Lives and Healthcare Costs in Maharashtra, India?
Source: Healthcare (Basel). 2023 Jul 24;11(14):2112. doi: 10.3390/healthcare11142112 (PMC10379405; doi:10.3390/healthcare11142112)

COVID-19 Pandemic: Did harsh mobility restrictions save lives and cost in Maharashtra, India?

Annexure-E

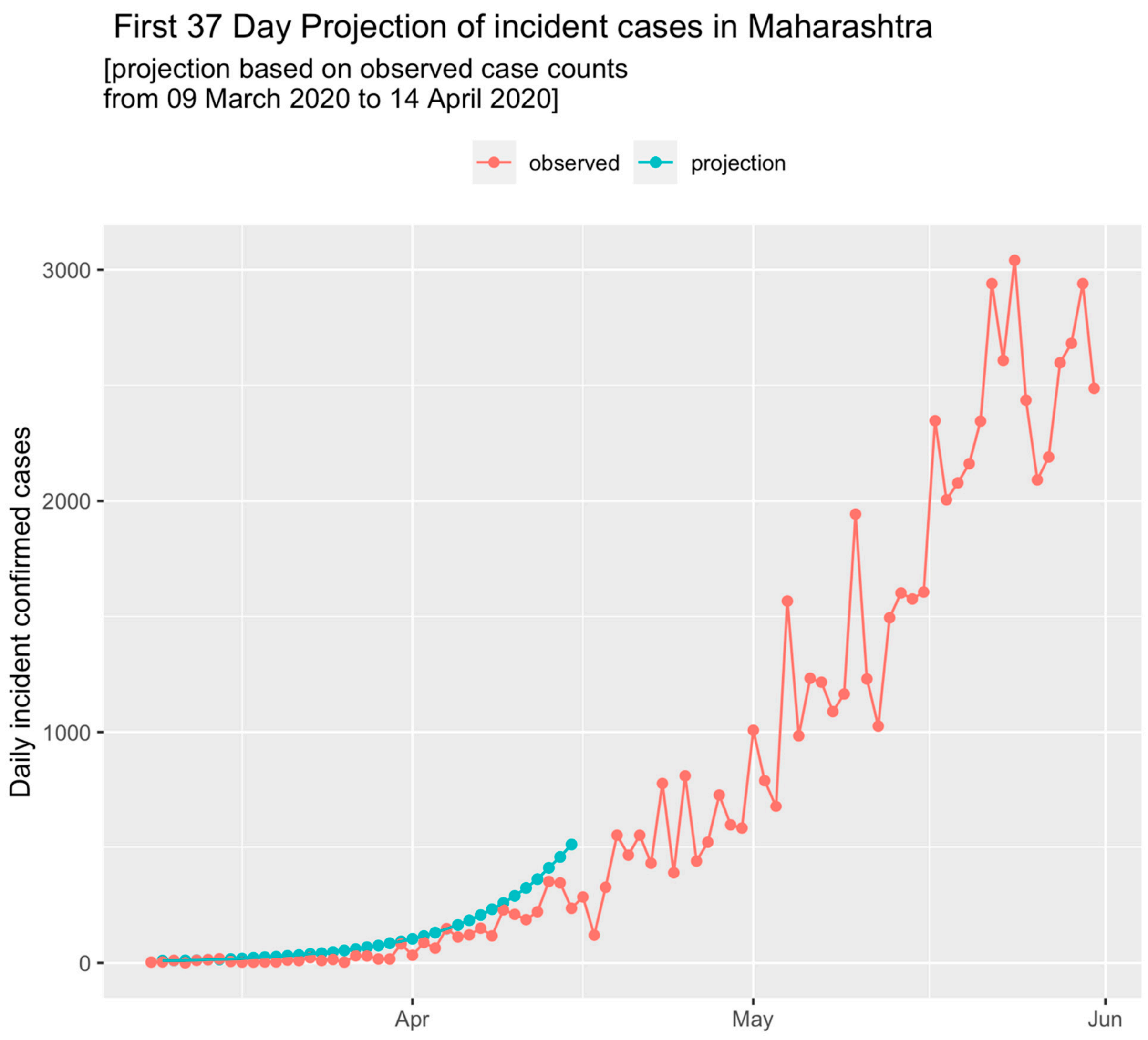

Supplement: Supplementary file 1 [file healthcare-11-02112-s001.zip › Ambade et al_2022_MH_COVID19_Annexure_SE.pdf]
